# Experiences of nurses and coordinators in a childhood obesity prevention trial based on motivational interviewing within Swedish child health services

**DOI:** 10.1080/17482631.2022.2096123

**Published:** 2022-07-15

**Authors:** Johanna Enö Persson, Christine Leo Swenne, Louise von Essen, Benjamin Bohman, Finn Rasmussen, Ata Ghaderi

**Affiliations:** a Division of Psychology, Department of Clinical Neuroscience, Karolinska Institutet, Stockholm, Sweden; b Department of Public Health and Caring Sciences, Uppsala University, Uppsala, Sweden; c Healthcare Sciences and eHealth, Department of Women and Children’s Health, Uppsala University, Uppsala, Sweden; d Centre for Psychiatry Research, Department of Clinical Neuroscience, Karolinska Institutet, and Stockholm Health Care Services, Region Stockholm, Stockholm, Sweden; e Department of Global Public Health, Karolinska Institutet, Stockholm, Sweden

**Keywords:** Child health services, qualitative research, paediatric obesity, primary prevention, motivational interviewing, nursing research

## Abstract

**Purpose:**

To explore the experiences of nurses and coordinators in the PRIMROSE childhood obesity prevention trial, and to understand the factors that might help to improve the outcome of future primary prevention of obesity.

**Methods:**

Using a qualitative approach, data were obtained by interviewing nine intervention nurses and three regional study coordinators. All participants were female. The interviews were transcribed and analysed using content analysis.

**Results:**

Two themes emerged: *The nurses experienced that it was rewarding to participate in the trial, but challenging to combine the intervention with regular work*; and *The study coordinators experienced that they were in a difficult position handling the conflicting needs of the research group and the nurses’ commitment to usual child health care services*. The importance of support, encouragement, briefer and simpler intervention, and adaptation of the training in motivational interviewing to the setting was emphasized. Stress and lack of time were major barriers to deliver the intervention as intended.

**Conclusions:**

Although the PRIMROSE intervention was developed in collaboration with representatives from the child health services, and additional research funding was provided to compensate for time spent working with the trial, nurses experienced stress and time constraints. .

## Background

Since the 1980s, the prevalence of overweight and obesity among children has been growing rapidly worldwide (Ng et al., 2014) and childhood obesity is considered to be one of the most serious health concerns of the 21^st^ century (WHO. Global Strategy on Diet, Physical Activity and Health, 2014). There are no recent national data for the prevalence of overweight and obesity among Swedish preschool children, but a regional study from 2016 showed that 10.7% of boys and 13.2% of girls were overweight at four years of age (Roswall et al., 2016). Childhood overweight and obesity is considered to be an important public health concern in Sweden, especially in areas with low purchasing power (Roswall et al., 2016).

Childhood overweight and obesity is associated with a wide range of health consequences of both psychological and physical nature (Gurnani et al., 2015; Sanders et al., 2015), as well as an increased risk of adult overweight and obesity (Simmonds et al., 2016). The importance of preventive initiatives has been emphasized, and a wide range of interventions has been developed and evaluated in different populations in a variety of settings (Wang et al., 2015; Waters et al., 2011). The results of systematic reviews examining randomized controlled trials with the goal to prevent obesity among preschoolers indicate only modest or no effect on objective measures, and questions regarding what works best for whom under what circumstances needs to be further investigated (Hunter et al., 2022; Peirson et al., 2015; Yavuz et al., 2015). In addition to controlled efficacy trials, studies that provide insights and generate hypotheses with regards to implementation and processes related to the lack of positive health outcomes might be a significant contribution to the body of knowledge (Johnson et al., 2022).

Qualitative methods are helpful to generate data with the purpose to formulate hypotheses for future research (Creswell, 2013). To the best of our knowledge, there are only a few studies exploring the views and experiences of people delivering parental interventions in obesity prevention trials. A systematic review of qualitative studies exploring facilitators and barriers to parents’ engagement and retention in parenting programmes concluded that participants, researchers and deliverers differ regarding the factors they consider the most important (Mytton et al., 2014). One study explored barriers experienced by nurses in Swedish child health centres (CHCs) when promoting healthy habits to parents (Ljungkrona-Falk et al., 2014). Four main barriers were identified: 1) barriers in the workplace, 2) the nurses’ fear and uncertainty, 3) obstacles in nurse—parent interactions, and 4) modern society impeding parents’ ability to promote healthy habits (Ljungkrona-Falk et al., 2014). Another study explored the experiences of teachers delivering an obesity prevention intervention in a primary school setting. The teachers reported positive experiences regarding the flexibility of the intervention and the ready-prepared materials, while time constraints and gaining support of parents were key challenges (Griffin et al., 2015). A qualitative study exploring the implementation of a childhood obesity prevention intervention targeting Swedish parents of six-year-olds emphasized the importance of clear information and well-functioning cooperation between project management, schools, and parents (Bergstrom et al., 2015).

The growing prevalence of childhood obesity in combination with the lack of knowledge on how best to address this public health concern (Brownell, 2010) highlights the need for more research exploring the experiences of those involved in the delivery of the interventions in obesity prevention research, in order to generate new knowledge for improving future interventions.

The current study was conducted within the PRIMROSE trial, a cluster-randomized controlled trial delivered to first-time parents at Swedish child health care centres (CHCs), with the aim of primary prevention of childhood obesity. The CHCs play a central role in health promotion and disease prevention for children from birth to school age (Bohman, Eriksson et al., 2013). More importantly, the CHC services are attended by almost all families regardless of ethnicity or socioeconomic status (Döring et al., 2014). The intervention was based on principles of cognitive behavioural therapy (CBT) and was delivered within the frame of motivational interviewing (MI). Parents and children in the control condition received care as usual (i.e., regular age-related health check-ups at CHCs). The PRIMROSE trial showed no effect in children in terms of physical activity (PA), waist circumference, body mass index (BMI; weight in kilograms divided by the square of the height in metres), or prevalence of overweight (including obesity). Small effects on parent-reported dietary habits of mothers and children were found, but the lack of effect on objective measures might indicate reporting bias (Döring et al., 2016; Enö Persson et al., 2017). The nurses who delivered the intervention had participated in extensive MI training, yet low levels of proficiency in MI were observed after the training workshop and subsequent supervision (Bohman, Forsberg et al., 2013; Eno Persson et al., 2016).

In the current study a qualitative approach was used to explore how participation in the PRIMROSE trial was experienced by two groups of nurses: those who delivered the intervention, and those who coordinated the trial regionally. Data was collected via semi-structured interviews. The primary aim was to explore the experiences of nurses who delivered the intervention and coordinated the study respectively. The secondary aim was to identify suggestions to be taken into account by researchers when planning future prevention studies.

## Methods

### Design

A qualitative research approach was applied to explore nurses’ and coordinators’ experiences of participation in a cluster randomized controlled trial of obesity prevention (Graneheim & Lundman, 2004).

### Setting

Details about the PRIMROSE trial, including study design, participants, training, methodology and content of the intervention have been published elsewhere (Bohman, Forsberg et al., 2013; Döring et al., 2016; Doring et al., 2014; Eno Persson et al., 2016; Enö Persson et al., 2017). The PRIMROSE trial was conducted in eight Swedish regions (former called counties) at 31 intervention CHCs and 28 control CHCs, between 2008 and 2015. In total 1355 families including 1369 children were enrolled (Döring et al., 2016), as well as 72 nurses working in the intervention group and 55 in the control group. Five regional study coordinators were part-time employed by the research team to help coordinate the data collection and the delivery of the intervention.

The participating CHCs were located in areas with diverse socio-demographics. First-time parents were invited to participate to promote their own, and their children’s healthy eating and physical activity habits for the purpose of primary prevention of childhood obesity. Parents were required to understand and speak Swedish to be able to participate, thus families who had rather recently migrated to Sweden were excluded. Details about the characteristics of the parents and children have been presented elsewhere (Döring et al., 2016; Doring et al., 2014).

The intervention was manual-based and consisted of nine sessions, of which six were delivered individually, two by telephone, and one in a group format. The duration of the intervention was approximately 39 months, and the families were enrolled when their child was nine months old. The sessions took place at the CHCs, often in conjunction with a regular health check-up, and otherwise as an additionally booked session. The check-ups are attended by almost all Swedish families and include vaccination, family counselling, and advice on child health and development. Parents generally met with the same nurse throughout the trial.

The aim of the intervention was to motivate parents to be healthy role models for their children and as the child grew older influence the child’s eating and PA behaviours (Doring et al., 2014). The intervention was delivered within the frame of MI, a client-centred, collaborative counselling style with the purpose to help motivating people for behaviour change by exploring and resolving ambivalence and eliciting change talk (the clients’ own arguments for change; Miller & Rollnick, 2002). (The spirit of) MI is (a mindset) characterized by collaboration, evocation, and autonomy/support, according to the 2002 version of MI that was used in the intervention (Miller & Rollnick, 2002). Evocation rests on the belief that people have an intrinsic motivation for change and that MI helps evoking it (Miller & Rollnick, 2002).

Before study-start, the nurses took part in a five-day workshop including lectures about the theoretical framework, nutrition, physical activity, and a 3.5-day training in MI. The study coordinators attended the workshop, although they weren’t actively engaging in the skills training. After the workshop the nurses received nine sessions of supervision on their MI skills, based on sessions that were audio-recorded and coded for proficiency in MI (Bohman, Forsberg et al., 2013; Eno Persson et al., 2016).

A central trial coordinator functioned as a link between the research group and the regional study coordinators, as well as the participating nurses, and helped coordinate the delivery of the intervention and data collection. The CHCs were given monetary compensation for their participation, which could be used to employ substitute nurses if needed.

### Participants

Nine nurses from eight CHCs across five counties and three study coordinators participated. At study-start, one to four years had passed since the nurses had delivered their last intervention session (and seven to eight years since the nurses had joined the PRIMROSE trial), thus a number of nurses had changed their workplace or retired. email addresses to 24 nurses still working at the CHCs were obtained from the study coordinators and all nurses were contacted by email twice. Some nurses who had not responded were strategically chosen and contacted by telephone (Malterud et al., 2016) in order to reach diversity in experiences regarding age, county and CHC. One nurse had retired and was not contacted initially, but a former colleague of hers informed us that she wanted to participate. Four nurses chose not to participate due to work-related stress, nine agreed to participate, and the remaining did not reply. Nine nurses were interviewed and nurses from five counties and eight CHCs participated. All participants were female and specialized as paediatric or district nurses, with a mean age of 47 years (*SD* = 9.7), and a mean number of years working at CHCs of 9.8 (*SD *= 8.1) when they joined the PRIMROSE trial.

The five regional study coordinators were contacted by email and three agreed to participate and two declined due to heavy workload. The participating coordinators were all female with specialist training, and were 47, 57, and 58 years old.

The number of participants was based on the extent to which the collected interview data provided information relevant to the aim of the study, e.g., the level of “information power” (Malterud et al., 2016). The number of subjects interviewed provided a high level of “information power”, no new information seemed to emerge from the last interviews (i.e., saturation emerged), and thus no further recruitment of subjects was needed. However, in qualitative research it is often difficult to know whether the studied phenomenon has been fully identified.

### Data collection

Data was collected from April to June 2016, using semi-structured face-to-face interviews conducted by the first author (JEP). Two interview guides (Appendix A) with open-ended questions including suggested follow-up questions were used. The questions were adjusted to fit the different roles of the intervention nurses and the regional coordinators. The questions were partly formulated based on an interview with the central coordinator of the trial, and partly based on discussions with nurses and regional coordinators at a symposium where the main trial results (Bohman, Forsberg et al., 2013; Döring et al., 2016; Eno Persson et al., 2016) were presented.

During the interviews, the participants were encouraged to reflect freely on their experiences and probes were used when further elaboration was needed. Before the interview, the participants received an email reiterating the aim of the interview and the key questions and they were asked to choose a time and location for the interview. All chose to be interviewed at their workplace during work hours. The length of the interviews ranged between 28 and 48 minutes. The participants were informed that they could contact the first author if they wanted to add anything that they did not mention during the interview. All interviews were audio-recorded and transcribed verbatim by a professional secretary. The recordings were saved on a secure server provided by the company that performed the transcriptions.

### Data analysis

The first author (JEP) performed the main part of the data analysis, in collaboration with the second author (CLS), and to some extent the third author (LvE), with feedback from the last author (AG). The transcripts were analysed using content analysis, a research method with the aim of reaching a condensed and broad description of a phenomenon (Elo & Kyngas, 2008; Graneheim & Lundman, 2004). Initially, JEP repeatedly read the transcripts to get an overall familiarity with the text, and as a next step identified meaning units. A meaning unit was defined as a text fragment that contained some information about the overall study aim (Malterud, 2012). The meaning units were subsequently condensed and coded, and from each code a category was created. An example of the analysis process from meaning unit to category can be seen in Table I. In the next step, the categories were collapsed into higher order categories based on similarities in meaning and content. These categories were then discussed back and forth, identifying similarities and dissimilarities and collapsing related categories into higher order categories (still called categories in Table II, while lower order categories are called Central characteristics of the category), until satisfactory agreement was reached. The meaning units and categories from the nurses’ and coordinators’ transcripts were analysed separately. In the next step two themes, i.e., an expression of latent content of the text or an underlying meaning of the meaning units, codes or categories (Graneheim & Lundman, 2004), were identified. Nurses’ advices to someone planning a trial similar to PRIMROSE were categorized (Elo & Kyngas, 2008; Graneheim & Lundman, 2004) and summarized.

**Table I.** Example of the analysis process.

Meaning unit	Condensation	Code	Category
I think that then I became scared. Then we all probably became pretty scared and after that there was a group that dropped out. I shouldn’t say scared but I was like “oh shit, what have we gotten ourselves into”. Yes, but then maybe we realized that this is pretty big, a bit bigger than we had expected. Or involve more work than we had expected.	Then we all probably became pretty scared and after that a group dropped out. I shouldn’t say scared but like “oh shit, what have we gotten ourselves into”. Maybe we realized that this was a bit bigger or involve more work than we had expected.	We felt “shit, what have we gotten ourselves into” realized it would involve more work than we had expected.	Worried when realizing that the trial would involve more work than expected, and believed others felt the same.

**Table II.** The nurses’ experiences of participating in the PRIMROSE trial.

Theme	Category	Central characteristics of the category
The nurses experienced that it was rewarding to participate in the trial, but challenging to combine working with the intervention with regular work at the CHC*	The training was appreciated but some parts were difficult and not related to working at CHCs	The CBT**-principles were hard to understand and remember
		The workshop and supervision was rewarding, to learn MI*** was most appreciated
		MI was hard to learn, the training was not related to working at CHCs
		Too much time passed between the workshop and the first MI session—had time to forget
	Not enough time to work with the trial	Realized the magnitude of the workload connected to the trial, and became worried
		A lot of work, time consuming, hard to make time which created stress
		Own responsibility for time management
		The CHC managers thought it took too much time
		Substitute nurses facilitated the work, but were not always available
		Negative influence on the relationship to colleagues who became more stressed
	MI hard to combine with work and tasks at the CHCs	Harder to use MI with already motivated parents
		The sessions hard to deliver, children and mandatory work assignments were distracting
	The manual was too extensive and complex	Helpful but also too extensive and difficult to understand
		MI hard to combine with detailed manual
	The parents’ schedule and priorities collided with the setup of the intervention	The parents thought that the trial took too much time, hard to make them come to the sessions
		Hard to get enough parents to come to the group sessions
		The parents’ wish to receive advice collided with MI
	The parents were interested and grateful, but it was hard to reach the ones who needed the intervention the most	Parents who would have needed the intervention the most declined participation, shame connected to weight was an obstacle
		Interested parents, the group session was appreciated
		Parents with low socioeconomic status benefitted less from the intervention
	Feelings of performance anxiety, unfamiliarity and shame	Anxiety and discomfort before the intervention sessions
		Discomfort before the supervision in the beginning
		Unfamiliarity with the technology of the recording devices created stress
		The recordings created performance anxiety
		Felt unnatural to use MI, used to giving advice
		Shame connected to being overweight herself
	Limited contact with the research group	Would have wanted more encouragement and recognition from the research group
		The research group was insufficiently informed about CHC work procedures
		Limited contact with the research group
	The importance of being heard and supported	The importance of the support from colleagues when facing obstacles
		The supervision was constructive, felt strengthened and understood
		The study coordinators gave practical and emotional support
		Positive CHC manager offered support
		Suggestions for improvements were listened to and implemented by the research group
	Sense of meaning, personal growth and responsibility	A lot of work but rewarding, could use parts of the new knowledge and skills in the clinical work
		Stayed in the trial due to a sense of duty
		The intervention sessions were positive, gained good relationships with the parents
		MI helped clarify the parents’ own ability and responsibility

*CHC, child health care centre

**CBT, cognitive behavioural therapy

***MI, motivational interviewing

## Results

The results section is divided into two sections. The first describes the results of the interviews with the intervention nurses (Table II), and the second describes the results of the interviews with the study coordinators (Table III). The results in the tables are organized in terms of themes, categories and central characteristics of the categories. In the text below, the results are presented using the themes as headings and the categories as subheadings in italics embedded within the text, following the same order as presented in the tables. Finally, the nurses’ and coordinators’ suggestions for future similar trials are presented in Table BI.

**Table III.** The coordinators’ experiences of participating in the PRIMROSE trial.

Theme	Category	Central characteristics of the category
The study coordinators experienced that they were in a difficult position handling the conflicting needs of the research group and the nurses’ commitment to usual child health care services	The nurses appreciated the MI* training but had difficulties learning the method	The nurses experienced the training in MI as rewarding
		Had the impression that the nurses had difficulty learning the more advanced MI skills
		Many nurses appreciated MI but there was a great diversity in engagement and development
	Not enough time to work with the trial	Realized how much work participation in the trial would involve for the nurses and became worried
		Had the impression that the nurses didn’t have time to deliver the intervention as it was intended
		Had to support the nurses and help them do their tasks
		Experienced that there wasn’t enough time allocated to the coordinator duties
	The intervention was not sufficiently adapted to the work at CHCs**	The nurses had difficulty focusing because of disturbing children and mandatory CHC work
	The manual was too extensive and complex	The manual was too extensive and the nurses had difficulty understanding parts of it
	Hard to reach the parents who needed the intervention the most	Parents who would have needed the intervention the most declined participation or dropped out
	The nurses experienced performance anxiety	The nurses had performance anxiety connected to recordings and supervision, the MI sessions felt unnatural
	Limited contact with the research group	The research groups’ lack of clarity on how to solve practical problems caused distress
		Stuck between the nurses’ and the research group’s conflicting needs
		Hard to be listened to and get help from the research group
	The importance of being heard and supported	The contact with other coordinators offered a great support
	Sense of meaning, engagement and responsibility	Continued working as a coordinator due to sense of responsibility
		Positive to get to know and be able to support the nurses
		Meaningful and educational to contribute to the development of the child health services

*MI, motivational interviewing

**CHC, child health care centre

### The nurses experienced that it was rewarding to participate in the trial, but challenging to combine working with the intervention with regular work at the CHC

Before the start of the intervention, the nurses took part in a five-day workshop/training. *The training was appreciated but some parts were difficult and not related to working at CHCs*. Nurses reported challenges with understanding and remembering the section about CBT principles and although most considered the training in MI and the subsequent supervision both fun and rewarding, learning the more complex MI skills were challenging, and a need for more examples related to working at CHCs was expressed.

There were a lot of references to treatments of addiction and smoking and such, so it was … maybe in the beginning … it was hard to connect it to our work, because there weren’t that many examples.

An additional challenge was the long time period between the workshop and the first intervention session, which made it hard to be on top of acquired knowledge and skills.

Most nurses found the work with the trial to be stressful and time-consuming, and felt that they had *not enough time to work with the intervention*.

You worked really hard, you really did. You booked as many families as you possibly could and every time you were on holiday you were punished in the sense that you had to work even harder and squeeze in more families when you came back.

The nurses mainly managed their time themselves, without the involvement of the CHC’s manager, yet in some cases the manager was critical of the amount of time that working in the trial took from regular work duties. The nurses reported perceived differences in workload at the different CHCs and some experienced high levels of stress. Stress was greatly influenced by the availability of substitute nurses, and although the CHCs received monetary compensation from the project to be able to hire substitutes, this was not always possible.

There was the opportunity to employ a substitute, but it was hard … in these kinds of small communities … then you’re supposed to find someone who … who can just step in and do this job.

In addition to the time constraints, the nurses reported several difficulties related to delivering the intervention to the parents, indicating that *MI was hard to combine with the usual work and tasks at the CHCs*. There were reports of difficulties using MI since most of the parents were already motivated to make any necessary changes or maintain an already healthy lifestyle, which presented few opportunities to use MI skills to solve ambivalence about change. Most nurses reported problems delivering the intervention when the children got older and constantly interrupted the conversations. They also found it hard to combine the intervention with mandatory work duties related to the regular health check-ups.

You’re supposed to follow this manual, and have this MI-conversation, and then you have this little creature who in fact is the main character in there … and during the whole time you’re supposed to do a developmental assessment of the child who might also be a disturbing element …

It was indicated that *the manual was too extensive and complex*, which sometimes made it hard to understand and use, for the nurses. The manual was also sometimes hard to combine with the use of MI.

An impression of some nurses was that *the parents’ schedule and priorities were in collision with the setup of the intervention*: parents reported distress and unwillingness to spend extra time at the CHCs to answer the same set of questions, especially when the child grew older and both parents were back on full time work. It became more and more difficult to make the parents come to the sessions and to complete their assignments. It was also difficult to attract a large enough number of parents to the group sessions, and at some CHCs there where simply not enough intervention families to create a group. According to the nurses some parents expressed frustration since they were expecting to get information and answers to questions about the child, which were in contrast with the MI approach. One nurse summarized her perceptions of a parent’s feelings as follows:

If I seek help for my child and ask for an advice, then I want an advice, not a reflection back.

According to some nurses, *the parents were interested and grateful, but it was hard to reach the ones who needed the intervention the most*. Some parents who were at heightened risk in terms of own overweight or obesity or unhealthy habits, declined participation or seemed to have felt uncomfortable during the sessions. The nurses had the impression that some parents’ reluctance could have been related to shame, that parents who were overweight or obese were reluctant to be weighed each session, and answer questions about their eating habits. In one case the parent was motivated in the beginning, but the nurse got the impression that failure to keep up with the new and healthier routine caused shame and guilt, leading to avoidance and defensiveness.

Well, I had a couple where the mother was very overweight and … it was difficult. Because the first sessions … she was very … we were going to make changes and it was going to be so great and she was going to exercise and … like that. And then I guess things happened in her life and she didn’t feel so good anymore and she sort of dropped everything and felt reluctant to come to the sessions … and I sensed that she became defensive …

It was indicated that parents’ socioeconomic status (SES) might have influenced their participation. Nurses who worked with parents of high SES (urban parents with high education and income) perceived these parents as highly motivated. However, they had the impression that most of them already had healthy habits at the onset of the trial. One nurse reflected that the parents in the expensive neighbourhood where she worked had little time for the intervention, since they had to work a lot to afford their housing. Parents with low SES on the other hand (rural parents with low education and income) could experience difficulties understanding some of the content of the intervention, and the community offered few opportunities for sports and exercise.

Working with the trial presented challenges of a personal nature and evoked some negative thoughts and emotions connected to the delivery of the intervention, e.g., *feelings of performance anxiety, unfamiliarity and shame*. There were reports of anxiety before the sessions, especially if the session was going to be recorded and used for supervision, and some nurses experienced anxiety before the supervision sessions. In both cases, anxiety was connected to performance, and the sense of being evaluated and maybe not measuring up, especially at the beginning of the trial. There were also reports of distress related to handling the recording devices, because of lack of technical knowledge and skills.

During the intervention sessions, some nurses experienced that using MI was “unnatural” to them and this made them feel self-conscious, as they were used to provide advice and to speak freely with the parents.

It was hard. You were not supposed to ask “why”, but sometimes I accidently did. And you were supposed to practice using that scale from 1 to 10 … and you weren’t supposed to say “why not” but sometimes I did. It was so many things, I don’t think I used everything because it was so advanced … to be able to do that you have to train more so that it comes naturally.

Talking to parents about the importance of healthy habits, could invoke uncomfortable feelings in the nurse if she herself was heavily overweight. One nurse thought that it became apparent that she was not practicing the healthy behaviours she was advocating, and she felt like a hypocrite. She handled this by telling the parents that she obviously knew how hard it is to change habits.

Based on the nurses’ reports, there were indications of *limited contact with the research group*, and a need for more positive feedback and recognition for their hard work. Some nurses expressed that the research group was insufficiently informed about the work procedures at the CHCs. Others were more neutral, and simply stated that they had no relationship with the researchers and almost no contact with them, or that they responded to practical questions in a satisfactory way.

*The importance of being heard and supported* by others, who offered understanding for the stressors connected to being involved in the trial, was emphasized by most of the nurses. This kind of validation and support helped them to feel understood, less lonely and provided strength when facing obstacles. The ones who offered this support were mainly colleagues who were also involved in the trial, the supervisors, and the study coordinators.

I know that we were tired and worn out sometimes and that we felt stressed, but at least we could share that with each other.

In one case the manager at the CHC had a supportive function, and in another case the nurse felt that her suggestions for improvements were listened to and implemented by the research group.

Although participation in the trial involved a lot of work, it also gave a *sense of meaning, personal growth and responsibility*; nurses found it rewarding to acquire new knowledge and skills, which they could use in their regular practice. However, one nurse described staying in the trial as a personal sacrifice due to a sense of duty. Some expressed that the intervention sessions were fun and that they led to better relationships with the participating families, others said that the use of MI helped them to see the parents’ own capabilities and responsibilities.

The MI way of thinking has helped me a little to see things from another perspective, and the feeling that yes, these people (the parents) also have abilities, and maybe that makes me worry less for things … although I still can lie sleepless sometimes.

### The coordinators experienced that they were in a difficult position handling the conflicting needs of the research group and the nurses’ commitment to usual child health care services

The coordinators were present during the nurses’ training before the start of the trial, and they got the impression that *the nurses appreciated the MI training, but had difficulties learning the method*. According to the coordinators, the nurses enjoyed learning MI and specifically appreciated the supervision following the workshop. However, the coordinators had the impression that the nurses had some difficulty learning the more advanced MI skills, and there was a great diversity in level of engagement and development of proficiency during the period of supervision.

The coordinators expressed that the nurses experienced a lot of stress and that there was *not enough time to work with the intervention* and deliver it as intended. Working as a coordinator involved spending a lot of time managing the nurses’ worry and negativity, and reminding them about different tasks related to the intervention. One of the coordinators took over some of the nurses’ tasks to make sure they were done in time.

Sometimes I … as coordinator … did it myself to make sure it would be done and to spare them.

Sometimes the coordinators experienced stress themselves, and this was especially true when there wasn’t enough time assigned to working with the trial.

The coordinators listened to the nurses’ telling them about their experiences of delivering the intervention to the parents and got the impression that *the intervention was not sufficiently adapted to the work at CHCs*. The coordinators had the impression that the nurses had difficulties focusing on MI during the intervention sessions, because of disturbing children and mandatory work duties.

The coordinators also had the impression that some nurses thought *the manual was too extensive and complex*, and difficult to navigate and understand (especially the handouts to the parents).

They thought it was a bit messy to navigate the manual, and I myself had some trouble with it.

From listening to the nurses’ reports, the coordinators got the sense that it was *hard to reach the parents who needed the intervention the most*, since these parents were more likely? to decline participation in the trial or drop out. It was mainly parents who were already healthy and motivated who chose to participate, and the coordinators expressed that they became reinforced in their beliefs of themselves as “good parents”, for example, when they filled in the food diary.

The parents who participated were parents who already had very good preconditions, who thought it was a bit exciting, and who were already conversant with the problem (of unhealthy habits) and had thought about it a lot.

The coordinators had the impression that *the nurses experienced performance anxiety*, after listening to them expressing anxiety about recording the intervention sessions and the forthcoming supervision that was based on the recordings. The anxiety seemed to have to do with being self-conscious, and that using MI was perceived as “unnatural” to some of the nurses.

There was lot of focus on the fact that it was a research project, and now we should do this, and now I must say that. It was not a natural conversation but more like, now it’s important that I say and do the right things and remember what I’d learned.

The coordinators expressed *limited contact with the research group*, because of lack of clarity on how to solve practical problems, especially at the beginning, and as a consequence the nurses were given additional administrative assignments, which caused them distress and frustration. This in turn resulted in distress for the coordinators, who were trying to balance the conflicting needs of the nurses and the research group, since the nurses’ stress and frustration collided with the research group’s agenda of making sure the trial progressed according to plan.

It was a bit hard because you knew the nurses and their problems, and then you had the research team who … wanted to push the project in a certain direction. So in a way it was a conflict between those needs.

The coordinators found it hard to push the nurses to prioritize the trial when they knew that they didn’t have time for their regular work at the respective CHC. In addition, they got the impression from the meetings with the research group, that there was no room for their suggested changes to the setup. Two coordinators expressed regret that they hadn’t been more persistent in explaining the nurses’ or their own situation to the researchers.

Since the role as coordinator presented a lot of challenges, *the importance of being heard and supported* by others was emphasized. The meetings with the other coordinators were seen as important, as they offered support and understanding, and helped to handle the nurses’ apprehensions and frustration.

We were all struggling with the same problems. And our meetings were a great strength, a very positive part of the project … very rewarding.

In addition, the contact with the central study coordinator was very positive; she made the coordinators feel that someone cared about them and listened to them.

Participating in the trial also gave the coordinators a *sense of meaning, engagement and responsibility*. The sense of responsibility could be a burden, making one coordinator stay in the project due to a sense of duty towards the nurses and the managers at the respective CHC. But the coordinators also experienced that it was gratifying to get to know the nurses and to be able to help and support them in their struggles, and the work gave a sense of meaning in terms of personal development, increased knowledge and the feeling of making a contribution.

It felt good. I think that I had their (the nurses) trust … and I guess that was the feedback that I got, that “it’s thanks to you that it has worked”. So, I think I’ve been a support to them.

### The nurses’ and study coordinators’ suggestions for future trials

The results from the nurses’ and study coordinators’ suggestions are summarized in Appendix B in supplementary files. Both nurses and coordinators highlighted the need to conduct a *feasibility study* and achieve *better adaptation to the CHCs*, by for example, involving nurses working with families at CHCs from the beginning. Both groups also addressed the need to *make the intervention briefer/simpler*, with an easier manual including handouts that also immigrant families could understand, more group sessions, less questionnaires for the parents, more administrative help, and only focusing on families where the child’s weight curve was showing heightened risk for overweight. *Booster sessions in MI* were also suggested, as well as *more feedback from the researchers on the results from the trial*. Some nurses suggested *more positive feedback from the research team regarding the nurses’ work efforts in the trial*. One nurse emphasized the importance to *avoid causing parental shame or guilt*, by for example, making it clear in the written material that you can have healthy habits and still be overweight, or by excluding the measure of parental weight. Some nurses reflected about the role of society in the aetiology of childhood obesity, and that only focusing on CHCs would probably not be enough to solve the problem, hence to *combine with interventions in other arenas than CHCs, including policy changes*, would be desirable. The coordinators suggested that nurses needed *more meetings with colleagues also working in the trial for exchange of experiences*, and *better time management plan devised by the research team* (more margins and back-up plans if something would go wrong) *and more upfront communication with the nurses about the extent of work* the trial would involve.

## Discussion

The primary aim of the current study was to explore the experiences of the nurses and study coordinators who had participated in the PRIMROSE childhood obesity prevention trial. A secondary aim was to identify suggestions to be taken into account in future trials. In sum, the data-analysis revealed two themes: *The nurses experienced that it was rewarding to participate in the trial, but challenging to combine working with the intervention with regular work at the CHC* and *The study coordinators experienced that they were in a difficult position handling the conflicting needs of the research group and the nurses’ commitment to usual CHC services.*

There were similarities between the nurses’ and coordinators’ experiences, and the findings indicate several areas of potential improvement to consider when designing future trials.

### The sufficiency of the training

The nurses believed that the training might have benefitted from more examples from CHC work. Some mentioned that they would have needed more training to learn the CBT principles and master the MI skills, and that long time intervals between the workshop and the first MI session (and subsequent supervision) might have impeded skill retention. The coordinators reported that the nurses displayed diversity regarding level of engagement in the training and skill development.

Difficulties to learn MI, and to sustain the skills over time have been reported in other studies (Brobeck et al., 2011; Curry-Chiu et al., 2015; Ostlund et al., 2015). In a previous study exploring nurses’ experiences of using MI in primary healthcare, the nurses described practicing MI as demanding and requiring experience and extensive training, as well as a genuine personal interest in the method and adequate time with the client to enable lifestyle changes (Brobeck et al., 2011). A meta-analysis of the sustainability of MI skills after training showed eroding skills during a six month period if no post workshop training was included (Schwalbe et al., 2014). The nurses in the PRIMROSE trial received extensive post workshop training consisting of nine sessions of supervision based on recorded and coded sessions, but in many cases the first supervised session took place several months after the workshop. Nurses were asked to book the first supervised session with suitable families, and were reminded to do so by the supervisors. Notably, potential barriers explaining the delay between the workshop and the first training session were not brought up during the interviews. Both nurses and coordinators suggested additional booster sessions in MI as a strategy to enhance skill retention. However, given the extent of the supervision, additional booster sessions might have been superfluous if the supervision had been delivered in close connection to the workshop. Two studies have evaluated the PRIMROSE nurses’ MI proficiency levels after workshop and supervision, showing a generally lower proportion reaching beginning proficiency levels compared to similar studies with similar training packages (Bohman, Forsberg et al., 2013; Eno Persson et al., 2016). This might in part be explained by the long time-intervals between the workshop and the first MI session that was coded and supervised. However, other possible explanations can be the nurses’ experiences of disturbances from accompanying children during the intervention sessions, and difficulty detecting target behaviours since many parents were already motivated and/or already had healthy habits (the MI fidelity measure were based on recordings of these sessions) at study-start.

### Time constraints and stress

The nurses and coordinators reported stress and lack of time to work with the trial, that work ended up being more time consuming than anticipated. The workload at the CHCs and the unavailability of substitute nurses seem to have greatly influenced their level of stress. From the perspective of the coordinators, stress occurred when the nurses were not able to allocate time to work in the trial. During the planning stage of the trial, the research team received feedback on the content of the intervention from nurses with prior experience of CHCs, and as a response to their input the intervention was made briefer and simpler than was initially intended. The researchers had anchored the trial with managers and policy makers in the involved regions and offered monetary compensation to the CHCs, yet the lack of substitute nurses was not anticipated. However, even if substitutes had been available, the demand from the CHCs to be financially reimbursed by the research team for additional costs due to participating in research might have been perceived by nurses as a sign of lack of support from their regional health care organization to give research equal priority as the routine work. This perception may have impacted their stress level. Several qualitative studies exploring practitioners’ experiences of working in trials and delivering interventions in clinical practice identified barriers in terms of time constraints (Curry-Chiu et al., 2015; Hilliard & Brenner, 2016) and difficulties combining the intervention with regular work duties (role adjustment; Graves et al., 2016). Thus, the problems reported in the current study are not unique, but seem to be part of a more general concern. Support and prioritization of research projects within the CHC structure, instead of over-dependence on research funding, and allocating time to nurses who participate in research by timely recruitment of additional personnel would probably benefit long-term implementation and development of research-based practice. The intervention was adjusted to the needs and resources of CHCs and available funding in several steps before the start of the trial. Some of the major challenges in the PRIMROSE trial were unexpected organizational problems in CHCs, e.g., lack of additional staff to hire, and high level of stress among nurses. Further adjustments of the intervention after the start of the trial, as suggested by the nurses, would neither have been a methodologically sound approach, nor an adequate way of addressing organizational difficulties. Anticipating potential problems in discussion with the involved CHCs, and ensuring enough organizational support for the staff involved in research should be a future priority when planning similar studies.

### Challenges in delivering the intervention at the CHCs

The nurses had difficulties combining MI with mandatory tasks, and with accompanying children who continuously interrupted the conversations. In addition, some reported that MI was difficult to apply when the parents were already motivated to make healthy lifestyle changes or already maintained a healthy lifestyle. MI is a method aiming at strengthening the client’s motivation and commitment to behaviour change(Miller & Rollnick, 2013), and it has been suggested that MI works best with clients with low motivation (Hettema & Hendricks, 2010), and should be used with caution with highly motivated clients (Lindqvist et al., 2017). We cannot conclude that MI is unsuitable for primary prevention based on the results from this study, but maybe the MI training could be adapted to better fit the target group in future preventive trials, or other methods might be more efficacious. Given the potential bias due to dropout based on the experience of nurses in this trial (i.e., high motivation among parents with high socioeconomic status and low usefulness of MI, and dropout by parents who seem to need this intervention the most, i.e., those parents with low socioeconomic status and/or own overweight/obesity) future trials might consider more selective prevention strategies than primary prevention. Increased knowledge on epidemiology of parents, their socio-economic status and actual health habits may help facilitate tailoring prevention trials based on actual needs and risk factors. However, the impression of nurses should also be seen in light of empirical data from the trial (Döring et al., 2016). At baseline, 36.7% of the mothers in the intervention group and 38% in the control group were overweight or obese. More interestingly, investigating drop-out based on quantitative data, we found no significant pattern in drop-out for parents with overweight or obesity. For example, the percentage of mothers with BMI below 25 kg/m^2^ who dropped out was 16.7%, while the corresponding figures for mothers with a BMI between 25 and 30 kg/m^2^ was 16.2% and for mothers with BMI above 30 kg/m^2^ was 17.7%.

The difficulty of combining other tasks/treatments in clinical practice with MI has been discussed by the developers of MI (Miller & Rollnick, 2013). They recognize the challenges inherent in moving between different perspectives while reassuring treatment fidelity. They recommend integrating MI or the spirit of MI in the entire clinical practice, but using flexible movement between the different MI processes (engaging, focusing, planning and evoking; Miller & Rollnick, 2013). Evoking, which is more unique to MI, is appropriate when strengthening motivation for change, and thus should be used only when this is called for (Miller & Rollnick, 2013). Also, if the CHCs’ structure and organization to a greater extent allowed nurses to have additional sessions without accompanying children, this might facilitate the use of MI in this context.

The manual was by some nurses and coordinators perceived to be too extensive and somewhat difficult to navigate and understand, and hard to combine with the use of MI despite several iterations and input from nurses in the planning phase of the trial. A less stressful work situation could potentially have enabled the nurses to allocate more time to reading and understanding the manual and preparing for the sessions. Whether a simpler and briefer manual would be more feasible and efficacious remain an empirical question. The use of a manual in combination with MI has been suggested to interfere with the spirit of MI, distracting from truly focusing on the client (Lundahl et al., 2010), and has been shown to produce smaller effect sizes compared to MI not combined with a manual (Hettema et al., 2005). Since an intervention that consists of several methods cannot be fully standardized and delivered with fidelity without a manual, the challenges of using MI in combination with a manual should receive further attention in future research.

### Parents’ expectations and life circumstances affected their participation

According to the nurses, some parents perceived their participation in the trial as too time-consuming, especially when both parents were back at work after parental leave. A review of studies of participants’ experiences of taking part in interventions have reported similar findings of competing demands on parents’ time and resources as a barrier to participation (Mytton et al., 2014). There have also been reports suggesting that parents generally don’t consider diet or physical activity as their main concern for their child (Norman et al., 2015; Slater et al., 2010). The PRIMROSE intervention consisted of nine sessions, of which two were delivered by telephone, and fewer sessions might not be sufficient to induce behaviour change, but a future similar initiative could potentially benefit from being delivered during a shorter overall time frame (Stice et al., 2006). However, systematic reviews of randomized controlled trials with the aim to prevent early childhood obesity have shown no effect on weight-related measures (Monasta et al., 2011; Peirson et al., 2015; Yavuz et al., 2015), which highlights the need for more research investigating the underlying mechanisms associated with change, in order to develop more effective interventions (Holmes et al., 2018). Combining the interventions at CHCs with interventions in other arenas was suggested by some nurses, since obesity is a multi-factorial societal problem. In line with the nurses’ reflections, the World Health Organization states that curbing the childhood obesity epidemic requires sustained political commitment and collaboration of public and private stakeholders (WHO. Global Strategy on Diet, Physical Activity and Health, 2014).

According to some of the nurses, a few parents reacted negatively to the MI methodology, since they had expected advice on specific concerns. A qualitative study investigating teachers’ and parents’ experiences of taking part in a Swedish universal childhood obesity prevention trial based on MI, also reported that parents were confused by the MI method, and had expected more direct advice (Norman et al., 2016). However, MI does allow the counsellor to offer information and advice, if the client asks for it, or if the counsellor asks for permission first (and he or she avoids expressing unsolicited and directive expert opinion; Miller & Rollnick, 2013). Thus, future trials aiming to evaluate MI in a CHC setting might benefit from putting more emphasis on the role of providing advice during the MI training given the context within which the intervention is performed. The problem might also be avoided by integrating MI and the MI spirit in all of the clinical practice (Miller & Rollnick, 2013). Then the nurses could more easily and flexibly provide advice or act in an MI-manner depending on what helps the parents the most.

Based on the impression of the nurses, highly motivated and healthy parents to a greater extent agreed to participate and stayed in the trial, and shame connected to parents’ own overweight/obesity/unhealthy habits, as well as low SES, might have been barriers to participation. Findings from the trial (Döring et al., 2016) show that at baseline, 36.7% of the mothers in the intervention group and 38.0% in the control group were overweight or obese. In addition, at baseline 67% of mothers in the intervention group and 60% in the control group had relatively high (post-secondary) education. In the total Swedish population of mothers with one child, the corresponding figure is 54.%. A study of Swedish CHC nurses’ conceptions of overweight and obesity in children showed that the nurses perceived the subject to be sensitive and that parents were ashamed and reluctant to talk about it. They also had the impression that parents with financial strains and low education were harder to reach (Isma et al., 2012). It has been acknowledged that socially disadvantaged groups are harder to recruit and retain in clinical trials (Cui et al., 2015; Diderichsen et al., 2012) and childhood obesity studies present with additional challenges to recruitment and retention (Cui et al., 2015). According to the Medical Research Council (MRC) guidance for developing and evaluating complex interventions, it is important to understand the whole scope of effects and how they vary among participants and sites, in order to design more effective interventions and apply them correctly across groups and contexts (Craig et al., 2013).

### The importance of communication and support

Some nurses and coordinators described the communication with the research group as insufficient, for example, due to uncertainties about how to solve practical problems that came up after the trial had started (leading to more work and stress), and lack of positive feedback and recognition for the nurses’ hard work. The coordinators tried to balance the conflicting needs of the nurses and the research team, yet some coordinators had the impression that there was no room for their suggestions for improvements. Once the design and methods are established, significant changes are not an option in a randomized controlled trial. In the PRIMROSE trial, a series of minor process evaluations were conducted, as recommended by the MRC guidance (Craig et al., 2013). A more systematic testing of a condensed form of the intervention over a shorter time period might have helped to investigate the feasibility of the intervention and to find contextual factors that could help researchers understand how the intervention would work in the clinical setting.

Both nurses and coordinators emphasized the importance of being listened to and supported. They expressed their gratitude regarding the support they had received from other colleagues involved in the trial. The nurses also felt supported by the MI supervisors and the coordinators, and examples of being listened to by the research group and the CHC manager were mentioned. The importance of support has been described in studies of clinicians’ experiences of working in clinical trials (Curry-Chiu et al., 2015; Graves et al., 2016; Hilliard & Brenner, 2016). Higgins and colleagues (Higgins et al., 2010) argue that research quality is enhanced when nurses who engage in research projects are sufficiently supported and their contributions appreciated, in contrast to lack of involvement and acknowledgement which leads to decreased motivation. Based on the results from the current study, the nurses seem to have been sufficiently supported from colleagues, coordinators, and supervisors. However, it was indicated that the support from the research group could have been improved with more positive reinforcement for the nurses’ and coordinators’ efforts.

### Personal barriers and facilitators

The nurses described having experienced negative emotions during their participation in the trial, such as performance anxiety associated with the MI sessions and the supervision, and using the MI approach instead of their usual communication style of providing advice. Performance anxiety experienced by clinicians using MI in primary care has been described in other studies (Curry-Chiu et al., 2015; Ostlund et al., 2015), as well as the difficulty suppressing previously learned habits of providing information (Brobeck et al., 2011; Curry-Chiu et al., 2015; Ostlund et al., 2015).

There were reports of discomfort/shame if the nurse herself was overweight, making her feel uncomfortable when talking to the parents about healthy habits. Even if the shame connected to overweight/obesity is widely recognized, there’s a lack of studies exploring the role of practitioners’ experiences of motivating clients to healthy behaviours while being overweight/obese themselves.

Many nurses and coordinators expressed positive emotions when working with the trial, and despite the hard work, they described it as a meaningful and enriching experience, and the use of MI as a way of reducing stress by giving more responsibility to the parents. In another study, the primary care nurses experienced the use of MI for health promotion as enriching and stimulating in terms of learning the method, observing the response from the patients, and having more trust in the clients’ own abilities to find solutions (Brobeck et al., 2011). In a study of nurses trained in MI and CBT techniques to support self-management of type 2 diabetes, similar results were found (Graves et al., 2016). It is probably a common experience of people to feel some level of performance anxiety and difficulties changing previously learned behaviours. Although the nurses felt nervous about the feedback on the recorded sessions, most expressed that they felt supported and strengthened during and after the supervision. Future trials might consider exploring whether potentially unpleasant feelings connected to participation is hindering adequate delivery of the intervention.

### Limitations

One to four years had passed since the nurses in the current study had delivered their last intervention session in the PRIMROSE trial. The time passed might have impeded their recollection of some details of their participation in the trial. In addition, most of the nurses and coordinators had taken part in a symposium where the main results of the trial were presented, including the low MI proficiency scores, which might have had an impact on their reflections.

## Conclusions

The results point to a number of suggestions for future trials aiming to evaluate the use of MI in the context of primary prevention of childhood obesity within CHCs. The importance of support, encouragement and unambiguous communication was emphasized. The use of MI might be facilitated by further adaptation of the training to the specific setting, putting more emphasis on the flexibility of the method. Nurses’ experiences of stress and time constraints seem to have been major barriers for delivering the intervention as intended. Future initiatives to be embedded in routine CHCs might benefit from more systematic feasibility testing and piloting before start of the trial.

## Data Availability

The transcripts from the interviews analysed in the current study are not publicly available since individual privacy could be compromised.

## Appendix A.

**Interview guide—nurses**

Main questions are marked by*. Other questions are potential follow-up questions.

*Do you remember why you agreed to work within the PRIMROSE trial?

*Before the start of the trial, you took part in training in motivational interviewing, as well as lectures about cognitive behavioural principles, nutrition and physical activity. How did you experience the training?

How did you experience the intervention sessions with the parents?

How did you experience working with the manual?

How did you experience using motivational interviewing?

What is your perception of the parents’ response during the sessions?

Did you experience any difficulties during the sessions?

How did you experience recording some of the sessions?

*How did you experience the supervision?

*How did you experience the support for your engagement in the PRIMROSE trial at your workplace?

*How did you experience your contact with the research team during the progression of the trial?

*To sum up, what was your experience from working in the PRIMROSE trial, compared to your expectations before the start of the trial?

*Was there anything that you perceived to be especially challenging or rewarding?

Have you had use of anything you’ve learned in PRIMROSE in your regular work as a nurse?

*Would you like to give any advice to someone planning a similar trial?

**Interview guide—regional study coordinators**

*Do you remember why you agreed to work within the PRIMROSE trial?

*Before the start of the trial, the nurses took part in training in motivational interviewing, as well as lectures about cognitive behavioural principles, nutrition and physical activity. Did you also attend the training? How did you experience the training?

*How did you experience your work as a regional coordinator?

*How did you experience your contact with the research team during the progression of the trial?

*How did you experience the contact with the intervention nurses?

What was your impression of their experiences of working in the trial?

*To sum up, what was your experience from working in the PRIMROSE trial, compared to your expectations before the start of the trial?

*Was there anything that you perceived to be especially challenging or rewarding?

*Would you like to give any advice to someone planning a similar trial?

## Appendix B.

**Table BI.** Nurses’ and coordinators’ suggestions for future trials.

Nurses	Coordinators
• Pilot study, better adaptation to the CHCs^a^	• Pilot study, better adaptation to the CHCs
• Make the intervention briefer/simpler	• Make the intervention briefer/simpler
• Booster sessions in MI^b^	• Booster sessions in MI
• More feedback on the results from the trial	• More feedback on the results from the trial
• More positive feedback from the research team regarding the nurses’ work efforts in the trial	• More meetings with colleagues also working in the trial for exchange of experiences
• Avoid causing parental shame or guilt • Combine with interventions in other arenas than CHCs, including policy changes	• Better time management plan devised from the research team and more upfront communication with the nurses about the extent of work

^a^CHCs, child health care centres.

^b^MI, motivational interviewing.
